# Supplementary material for: The circulating cell-free DNA landscape in sepsis is dominated by impaired liver clearance
Source: Cell Genom. 2025 Aug 11;5(10):100971. doi: 10.1016/j.xgen.2025.100971 (PMC12790999; doi:10.1016/j.xgen.2025.100971)
Supplement: Document S1. Figures S1–S7 and Table S1 [file mmc1.pdf]

Cell Genomics, Volume *5*

Supplemental information

The circulating cell-free DNA landscape in sepsis is dominated by impaired liver clearance

Kiki Cano-Gamez, Patrick Maclean, Masato Inoue, Sakineh Hussainy, Elisabeth Foss, Chloe Wainwright, Hanyu Qin, Stuart McKechnie, Chun-Xiao Song, and Julian C. Knight

Supplemental Information

Supplementary Tables

Table S1. Definitions of clinical variables used in this study (related to STAR methods).

Clinical variable	Definition	Units
Albumin	Lowest albumin measured on the day of sampling.	g/l
ALT	Highest (i.e. ‘peak’) alanine aminotransferase (ALT) measured on the day of sampling.	IUL
AST	Highest (i.e. ‘peak’) aspartate aminotransferase (AST) measured on the day of sampling.	IUL
Bilirubin	Highest (i.e. ‘peak’) total bilirubin measured on the day of sampling.	μM
Blood cell counts	Highest cell counts per cell type group observed on the day of sampling.	1 x 10^6 cells/μl
Creatinine	Highest (i.e. ‘peak’) creatinine measured on the day of sampling.	μM
CRP	Highest (i.e. ‘peak’) C-reactive protein (CRP) measured on the day of sampling.	mg/l
Haematocrit	Lowest haematocrit measured on the day of sampling.	%
Haemoglobin	Lowest haemoglobin measured on the day of sampling.	g/l
INR	International normalised ratio calculated from the highest (i.e. ‘peak’) prothrombin time (PT) measured on the day of sampling.	-
MAP	Lowest mean arterial pressure (MAP) measured on the day of sampling.	mmHg
NLR	Neutrophil-to-lymphocyte ratio (NLR) calculated from the highest blood cell counts obtained on the day of sampling.	-

Supplementary Figures

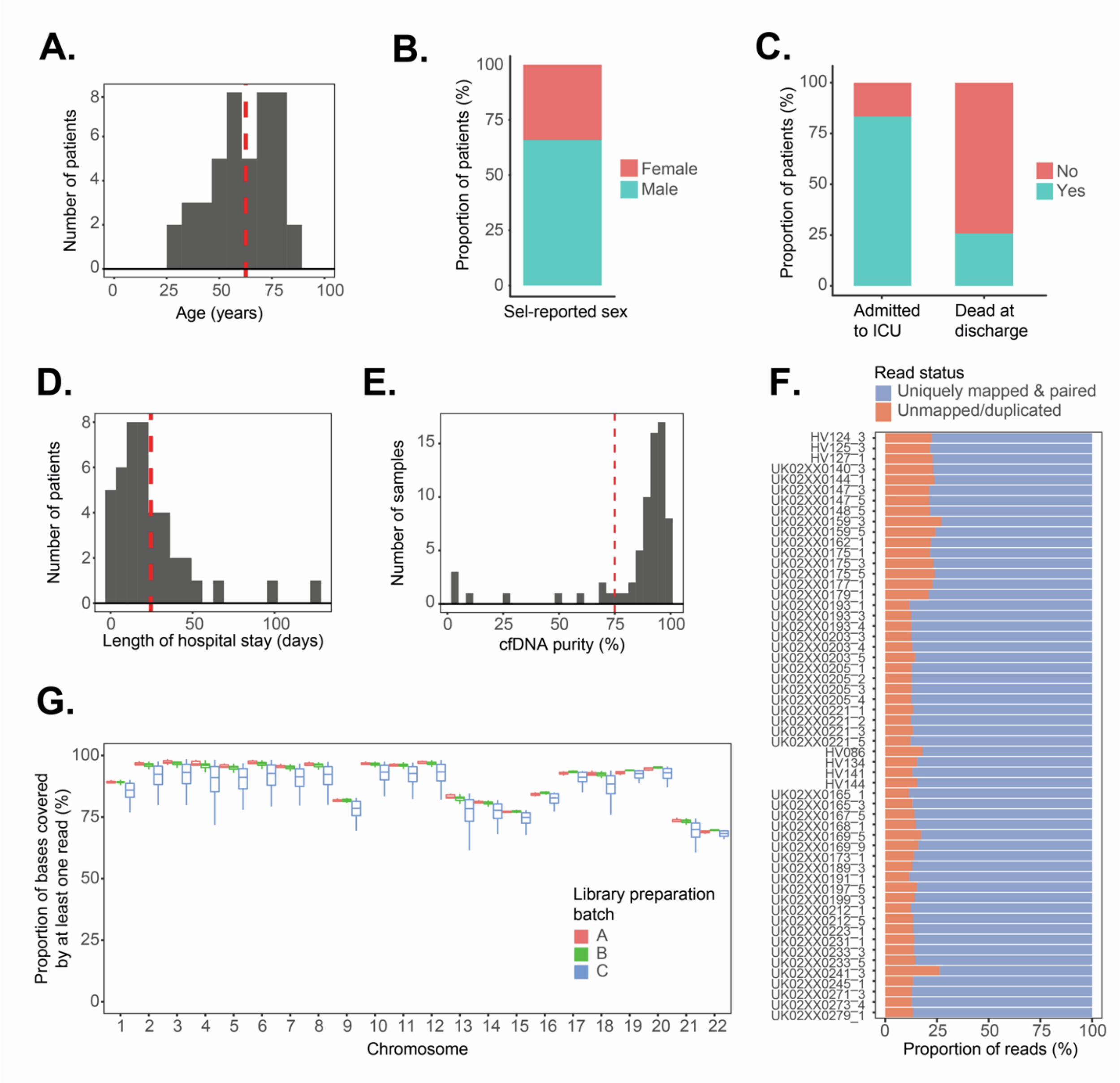

Figure S1. Cohort demographics and sample quality metrics (related to STAR methods and Figure 1). A. Distribution of self-reported age in our patient cohort (n=74 samples from 46 sepsis patients). The red dotted line indicates the mean age across patients. **B.** Distribution of self-reported sex in our patient cohort. Bar plots indicate patient proportions. **C.** Proportions of patients admitted to the ICU (left) and reported dead at hospital discharge (right). **D.** Distribution of hospital stay duration in our patient cohort. The red dotted line indicates the mean value. **E.** Distribution of cfDNA purity after isolation from plasma as estimated using capillary electrophoresis. The red dotted line indicates the cut-off 75% used for sample exclusion. **F.** Proportion of reads uniquely mapped to the human genome after sequencing in each sample in our study. **G.** Distribution of sequencing coverage in each chromosome is shown as the proportion of bases covered at > 1X (Y axis). Samples are stratified by TAPS library preparation batch. Box plots indicate the median and IQR for each sample.

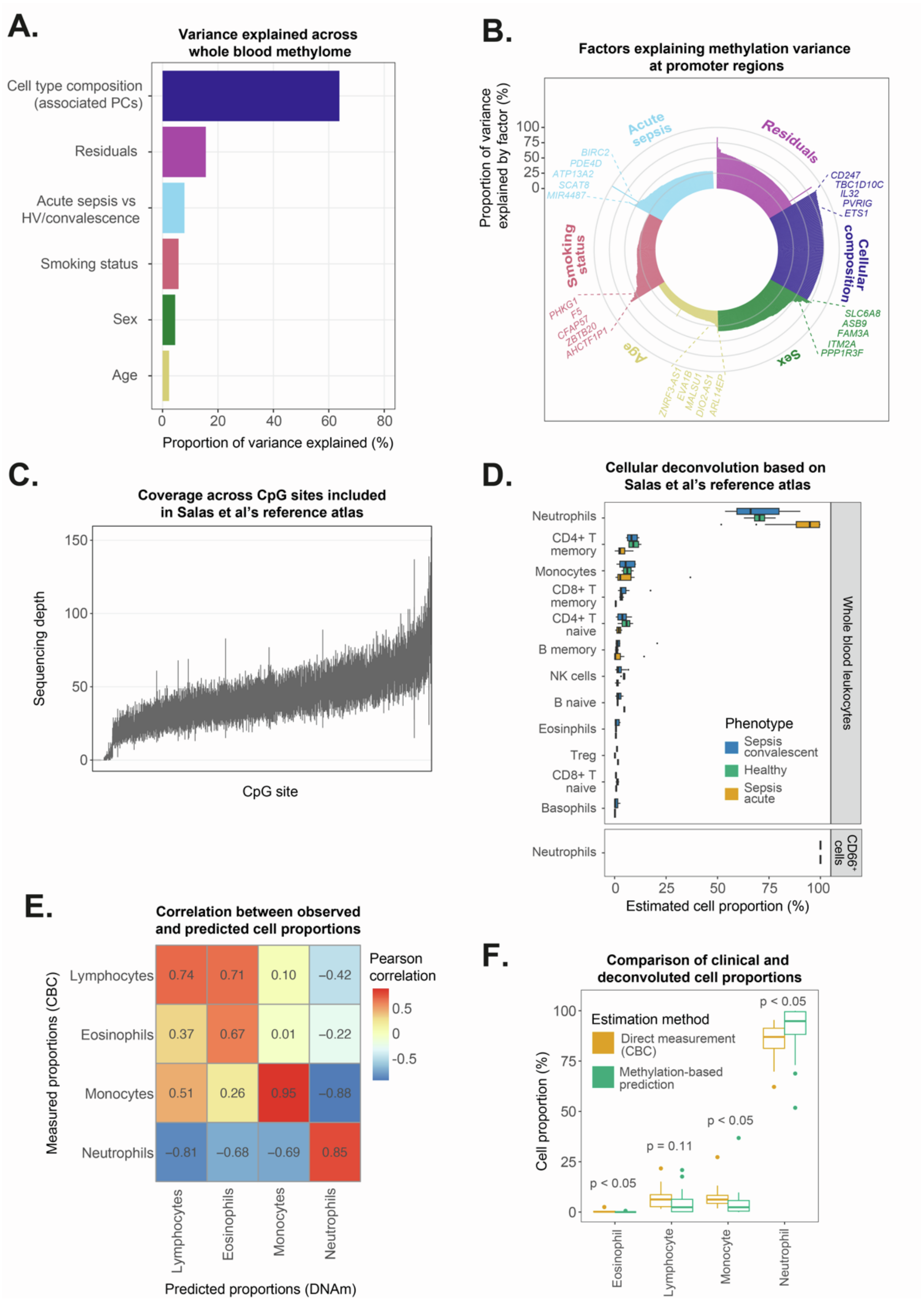

Figure S2. Analysis of the whole blood leukocyte methylome in 15 sepsis patients (related to STAR methods and Figure 4). A. The proportion of variance in WBL methylome (as profiled using EM-seq) (X axis) explained by different covariates (Y axis) was estimated using variance partitioning analysis. **B.** Variance partitioning estimates are shown for each CpG site included in the WBL methylation data set. The proportion of variance explained (Y axis) is shown for each assessed covariate (colour hue). Each bar represents a CpG site, with sites being ordered by decreasing variance estimate. **C.** Sequencing coverage (Y axis) achieved for each of the CpG

sites included in Salas's methylation tissue atlas (X axis). **D.** Cell type proportion estimates derived from WBL methylome deconvolution using EpiDISH and Salas's methylation atlas. Colours indicate sample groups (n=15 acute sepsis patients, n=5 healthy controls, and n=9 convalescent/recovered sepsis patients). Bar plots indicate medians and IQRs for each cell type in the reference atlas. **E.** Correlation between cell proportions estimated from WBL methylome deconvolution and directly measured in a complete blood count (CBC) in hospital. Colours indicate Pearson correlation coefficients, with rows and columns ordered by similarity using hierarchical clustering. **F.** Cell proportion estimates (Y axis) derived from WBL methylome deconvolution and CBC measurements (colour hue). Box plots indicate median and IQR values for each cell type.

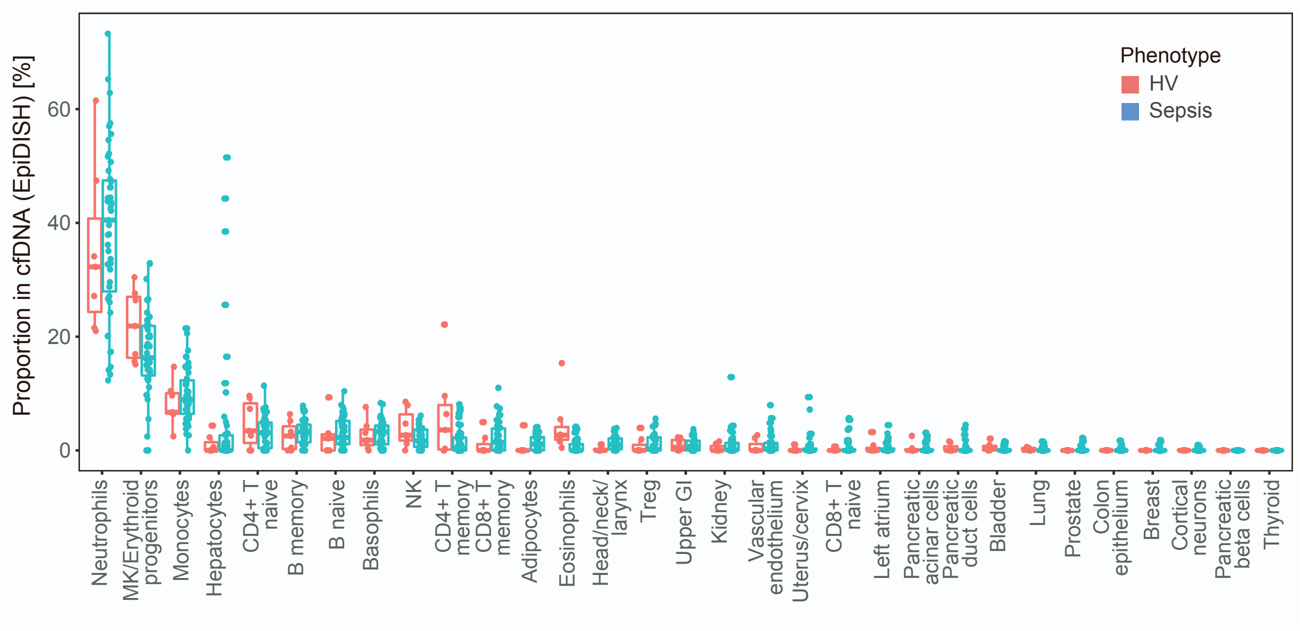

Figure S3. Cell-free DNA deconvolution based on Moss et al.'s methylation atlas (related to Figure 4). Proportion of cfDNA estimated to arise from different tissues based on methylome deconvolution using EpiDISH and the reference tissue atlas published by Moss et al. Box plots show median and interquartile ranges (IQR) of estimated proportions in sepsis patients (red) and healthy controls (blue).

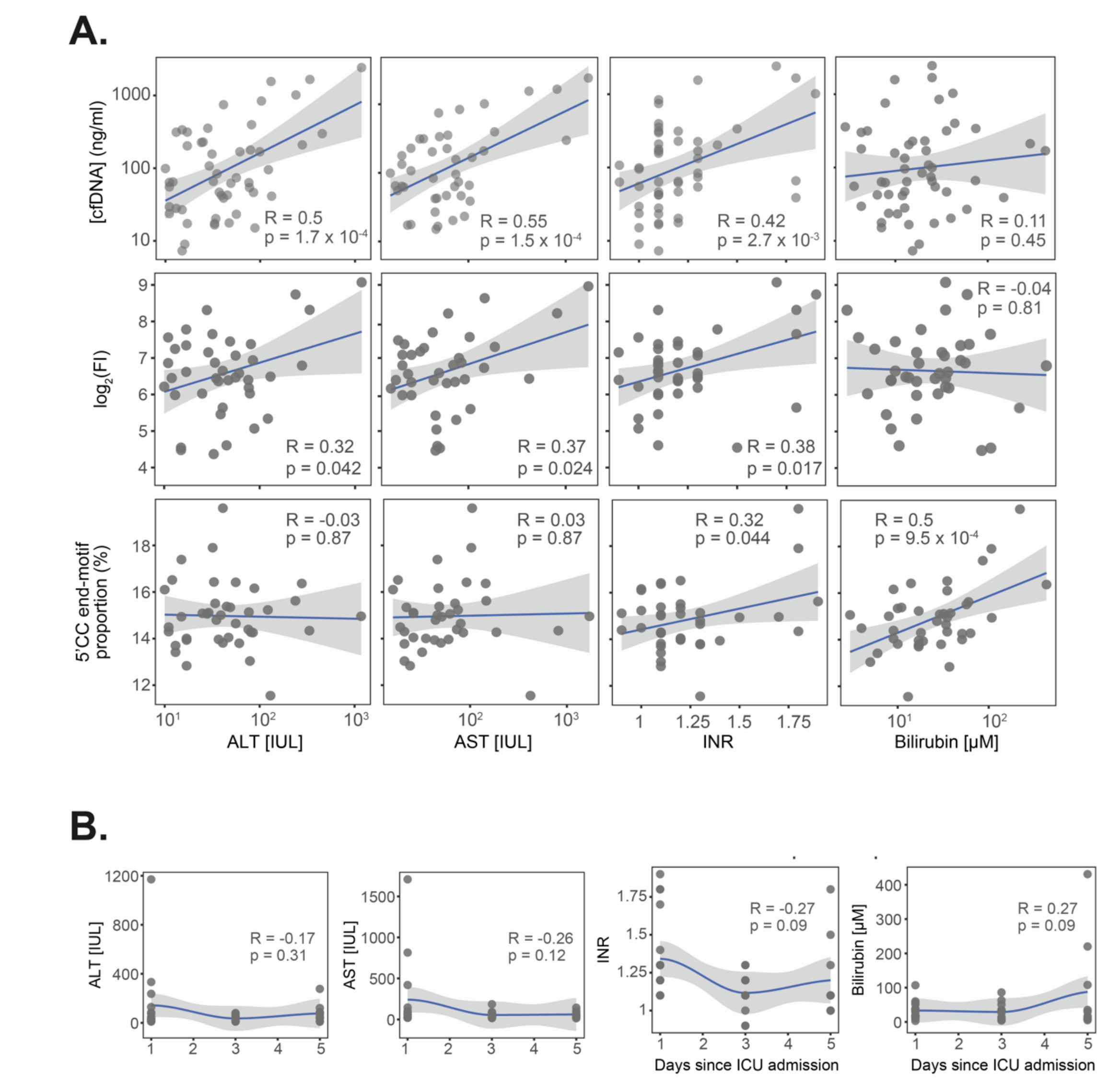

Figure S4. Relationship between cfDNA fragmentation features and liver function tests (related to Figure 5). A. Relationship between time since admission (X axis) and measurements for different clinical liver function tests (Y axis). Each dot represents a sample, with blue lines and shaded regions indicating fits and confidence intervals from locally estimated scatterplot smoothing (LOESS). Correlation coefficients and p values were estimated using Pearson correlation tests. **B.** Correlation between different clinical liver function tests (X axis) and cfDNA features (Y axis), including cfDNA concentrations in plasma (top row), cfDNA fragmentation indices (middle row), and frequencies of 5'CC end motifs (bottom row). Each dot represents a sample, with blue lines and shaded regions indicating linear fits and confidence intervals. Correlation coefficients and p values were estimated using Pearson correlation tests.

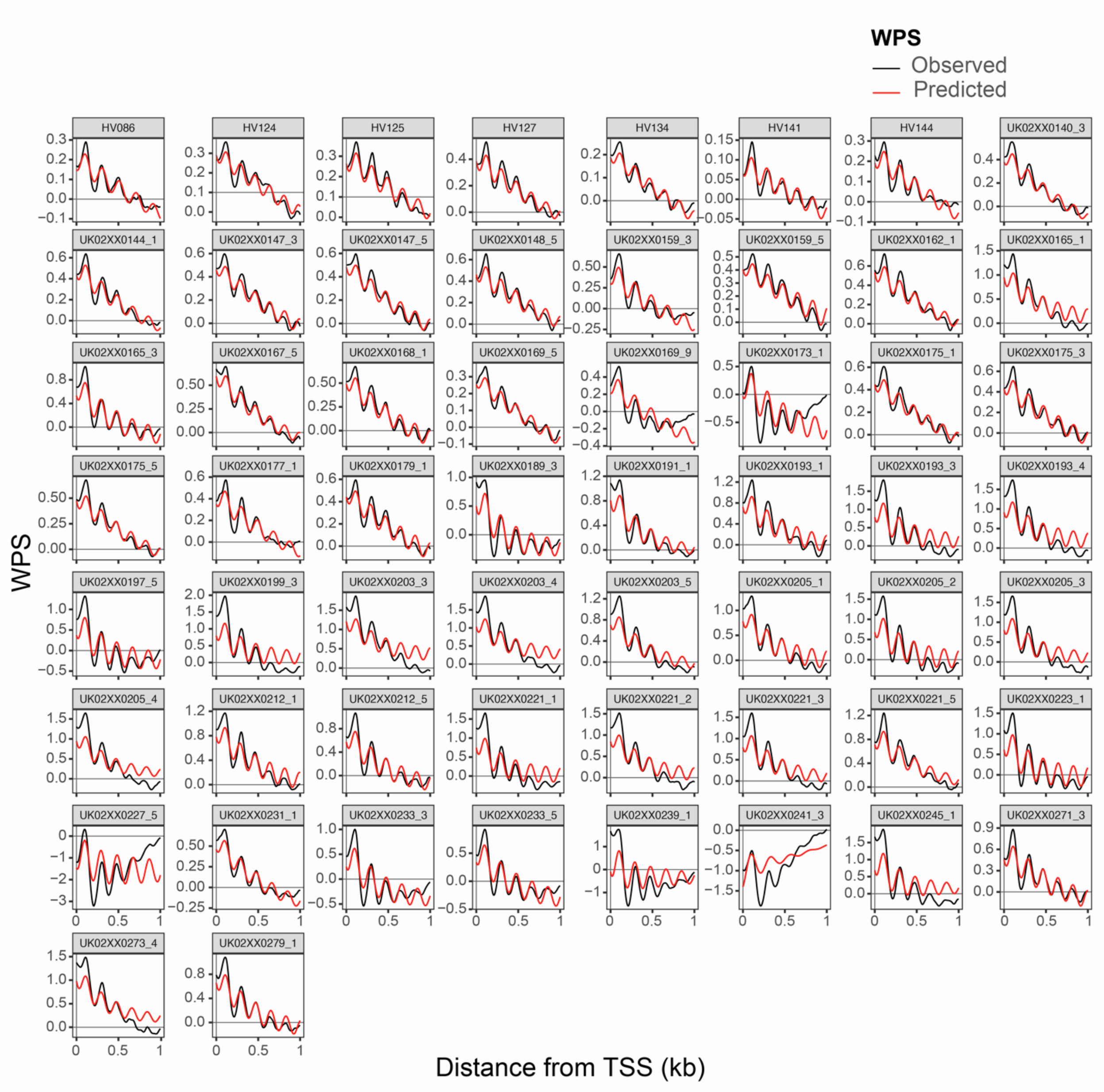

Figure S5. Dampened harmonic oscillator modelling of nucleosome phasing across samples (related to Figure 6). Observed WPS values at the TSS regions of all known genes (black lines) are shown alongside predictions derived from a dampened harmonic oscillator model (red lines) in each sample in our study. Model fitting was performed using non-linear least squares analysis. Each panel represents estimates from a different patient sample.

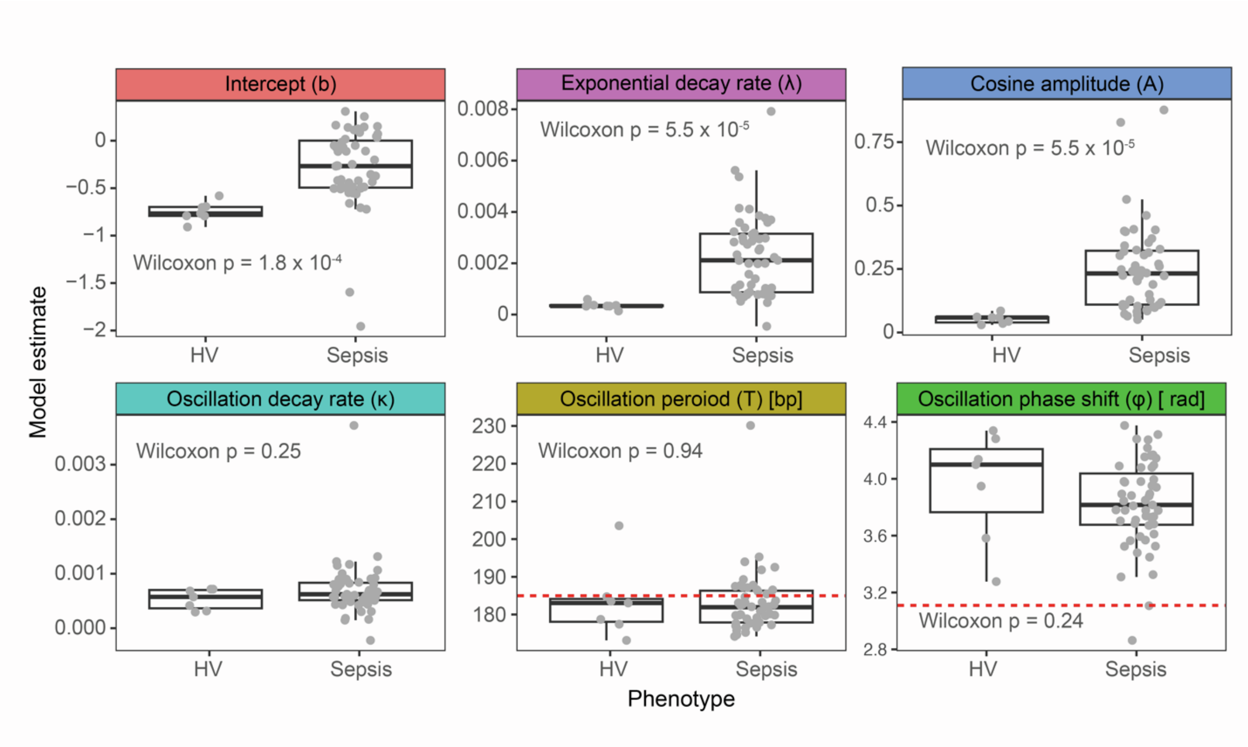

Figure S6. Nucleosome phasing parameter estimates (related to Figure 6). Parameter estimates from a dampened harmonic oscillator model were inferred using non-linear least squares analysis. Estimates (Y axis) are shown for each sample, stratified by disease status (X axis). Each panel shows estimates for a different parameter in the model equation. Dotted red lines indicate expected parameter values based on known biology. Box plots indicate median and IQRs for each parameter. P values were estimated using Wilcoxon rank sum tests.

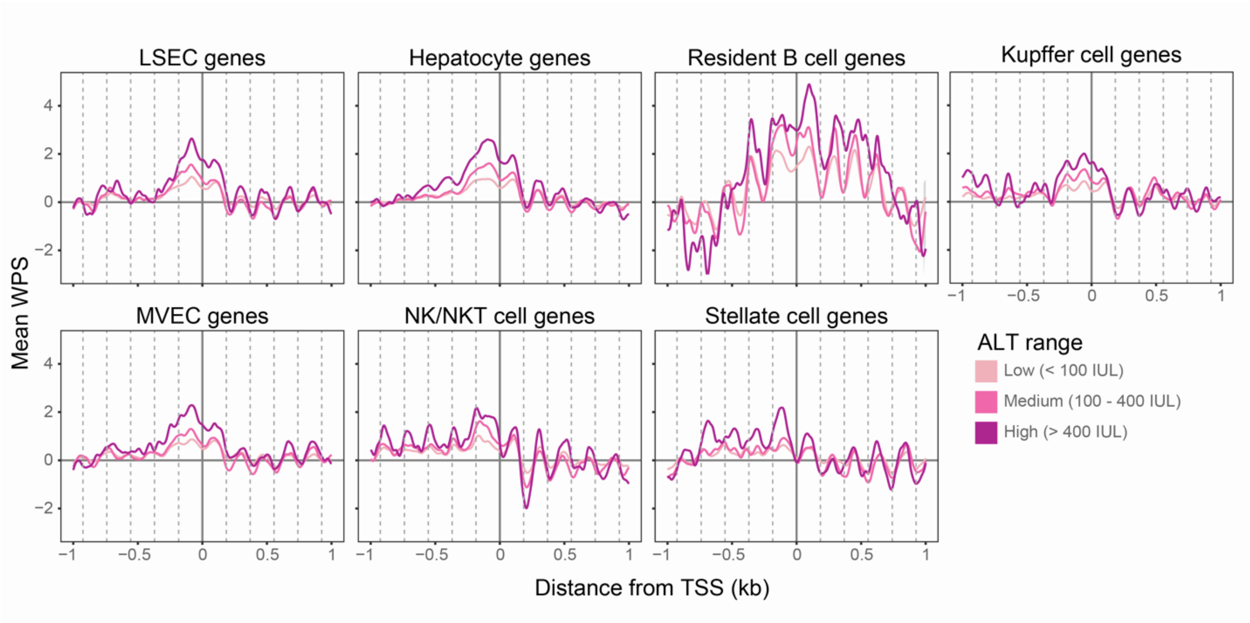

Figure S7. Nucleosome phasing strength at liver-specific gene sets correlates with liver dysfunction (related to Figure 6). Average WPS values at the TSS regions of genes specifically expressed in a variety of liver cell types, as determined by Aizarani et al.'s liver cell atlas. Lines indicate the average WPS values across samples classified as high, medium, or low levels of circulating ALT (colour hue). Dotted vertical lines indicate the expected position of nucleosomes around the TSS.
